# The association between EAE development in mice and the production of autoantibodies and abzymes after immunization of mice with different antigens

**DOI:** 10.1111/jcmm.16183

**Published:** 2021-02-09

**Authors:** Kseniya S. Aulova, Andrey A. Urusov, Sergey E. Sedykh, Ludmila B. Toporkova, Julia A. Lopatnikova, Valentina N. Buneva, Sergei V. Sennikov, Thomas Budde, Sven G. Meuth, Nelly A. Popova, Irina A. Orlovskaya, Georgy A. Nevinsky

**Affiliations:** ^1^ Institute of Chemical Biology and Fundamental Medicine Siberian Branch of the Russian Academy of Sciences Novosibirsk Russia; ^2^ Institute of Clinical Immunology Siberian Branch of the Russian Academy of Sciences Novosibirsk Russia; ^3^ Institut für Physiologie I Westfälische Wilhelms‐Universität Münster Germany; ^4^ Department of Neurology Westfälische Wilhelms‐Universität Münster Germany; ^5^ Institute of Cytology and Genetics Siberian Branch of the Russian Academy of Sciences Novosibirsk Russia

**Keywords:** abzymes, autoantibodies, C57BL/6 mice, colony formation, DNA complexes with proteins, EAE model, haematopoietic progenitor differentiation, immunization with MOG

## Abstract

We have previously shown that immunization of C57BL/6 mice, prone to spontaneous development of experimental autoimmune encephalomyelitis (EAE), with three antigens (MOG_35‐55_, DNA‐histone complex or DNA‐methylated BSA complex), alters the differentiation profiles of bone marrow haematopoietic stem cells (HSCs). These are associated with the production of autoantibodies (auto‐Abs) against these antigens and the formation of abzymes hydrolysing DNA, MOG, myelin basic protein (MBP) and histones. Immunization of mice with antigens accelerates the development of EAE. This work is the first to analyse the ratio of auto‐Abs without and with catalytic activities at different stages of EAE development (onset, acute and remission phases) after immunization of mice with the three specific antigens. Prior to immunization and during spontaneous in‐time development of EAE, the concentration of auto‐Abs against MBP, MOG, histones and DNA and activities of IgG antibodies in the hydrolysis of substrates increased in parallel; correlation coefficients = +0.69‐0.94. After immunization with MOG, DNA‐histone complex or DNA‐met‐BSA complex, both positive (from +0.13 to +0.98) and negative correlations (from −0.09 to −0.69) were found between these values. Our study is the first showing that depending on the antigen, the relative amount of harmful auto‐Abs without and abzymes with low or high catalytic activities may be produced only at onset and in acute or remission phases of EAE. The antigen governs the EAE development rate, whereby the ratio of auto‐Abs without catalytic activity and with enzymatic activities of harmful abzymes hydrolysing MBP, MOG, histones and DNA varies strongly between different disease phases.

## 1 INTRODUCTION

Antibodies (Abs) against chemically stable analogs of transition states of chemical reactions and natural antibodies with catalytic activities (abzymes) are well‐described in the literature (for review, see refs. 1, 2, 3, 4, 5, 6). IgG and/or IgA and IgM (immunoglobulin G, A and M) antibodies that hydrolyse DNA, RNA, adenosine triphosphate (ATP), polysaccharides, peptides and proteins were found in the blood of patients for various autoimmune pathologies (AIPs), including systemic lupus erythematosus (SLE), multiple sclerosis (MS), Hashimoto's thyroiditis, polyarthritis, lymphoproliferative diseases, polyneuritis and malignant tumours, as well as for three viral diseases—viral hepatitis, tick‐borne encephalitis and human immunodeficiency (Refs. 1, 2, 3, 4, 5, 6 and references therein). In contrast, antibodies from healthy donors or from patients with diseases without significant impairment of the immune status do not show significant catalytic activities.^1^, ^2^, ^3^, ^4^, ^5^, ^6^

Autoantibodies (auto‐Abs) to various antigens are formed both in healthy donors^7^, ^8^ and in patients with AIPs.^1^, ^2^, ^3^, ^4^, ^5^, ^6^ In patients with multiple sclerosis (MS), auto‐Abs against DNA were found in elevated concentrations in only 17%‐18%^9^ and abzymes with DNase activity in 90%‐95% of patients,^10^ compared with healthy donors. For patients with AIPs, the average values of antibody levels in comparison with healthy donors usually increase only in the late or worsening stages of the disease.^1^, ^2^, ^3^, ^4^, ^5^, ^6^ Different abzymes with various catalytic activities in healthy donors are usually either absent or show extremely low activities.^1^, ^2^, ^3^, ^4^, ^5^, ^6^, ^15^ In contrast to enzyme‐linked immunosorbent assay (ELISA), the catalysis of different reactions by abzymes is based on a large number of catalyst turns, even if they possess low activity. Therefore, different abzymes can be found even at the onset of AIPs.^1^, ^2^, ^3^, ^4^, ^5^, ^6^

The statistically significant appearance of abzymes can be detected in the earliest stages of various pathologies when changes in antibody levels to specific antigens (eg DNA, MBP, thyroglobulin) still correspond to the range of changes seen in healthy donors.^1^, ^2^, ^3^, ^4^, ^5^, ^6^ This was confirmed by the ratio of relative auto‐antibody concentrations to DNA and abzymes hydrolysing DNA during spontaneous SLE development in autoimmune MRL‐lpr/lpr mice^16^, ^17^, ^18^ and abzymes hydrolysing proteins during EAE development in C57BL/6 mice.^19^, ^20^, ^21^, ^22^ Thus, the appearance or strong increase (dozen‐fold or more) of abzymes’ relative activities (RAs) in relation to levels seen for healthy individuals can be one of the earliest signs of AIP development.^16^, ^17^, ^18^, ^19^, ^20^, ^21^, ^22^

It has been previously suggested that AIP development may be because of defects in haematopoietic stem cells.^23^ To investigate this issue, we have in previous studies used two different models of autoimmune prone mice: MRL‐lpr‐lpr (SLE model)^16^, ^17^, ^18^ and C57BL/6 (human MS model).^19^, ^20^ We analysed the differentiation profile of bone marrow haematopoietic stem cells (HSCs) during spontaneous and antigen‐stimulated development of SLE (DNA immunization) and EAE (immunization with MOG). We found that, compared with the norm, the spontaneous pre‐disease phase in SLE and EAE mice led to first changes, and transition to deep pathology led to additional changes in the differentiation profile of HSCs (ie BFU‐E, CFU‐E, CFU‐GM and CFU‐GEMM). During initial changes in HSC differentiation profile, abzymes hydrolysing DNA and MBP in the blood of mice showed only relatively low activity, whereas their activity increased greatly during later disease stages.^16^, ^17^, ^18^, ^19^, ^20^, ^21^, ^22^ Immunization of SLE mice with DNA accelerates disease development and being associated with an increase in antibody levels against DNA, as well as in the increase in RAs of abzymes hydrolysing DNA, ATP and oligosaccharides.^16^, ^17^, ^18^ After treating EAE mice with MOG, DNA‐histone complex or DNA‐met‐BSA complex, the first strong increase in antibody levels against MOG, MBP and DNA and in abzymes that hydrolyse these substrates was already observed after seven days; the maximum increase was observed 18‐20 days after immunization.^19^, ^20^, ^21^, ^22^ Notably, changes in HSC differentiation profiles were similar for SLE and EAE mice during spontaneous and antigen‐induced development of these pathologies.^19^ In parallel with the differentiation profile change for HSCs, the relative number of lymphocytes producing auto‐Abs and abzymes increased.^19^, ^20^, ^21^, ^22^

Interestingly, the RA of abzymes derived from the cerebrospinal fluid of MS patients, hydrolysing MBP, DNA and oligosaccharides depending on their substrate, is about 40‐60 times higher than Abs found in the blood of the same patients.^24^, ^25^, ^26^

Analysing the development of SLE and EAE showed that the appearance of abzymes is the earliest and also a statistically significant marker for the development of these AIPs. A further significant increase in abzyme activity correlates with additional changes in the differentiation profile of bone marrow stem cells, which associated with increases in urine protein concentration, anti‐DNA and anti‐protein Abs in the blood and leads to the appearance of a typical pink butterfly in SLE mice and alopecia in EAE mice.^16^, ^17^, ^18^, ^19^, ^20^, ^21^, ^22^

Accelerating EAE development in C57BL/6 mice after MOG immunization is a classic model for studying possible patterns of MS development in humans. However, until recently, there was no evidence whether only MOG and MBP induce the development of EAE in mice and MS in humans or whether other immunogens also have similar properties. Histones and their post‐translational modifications play a key role in chromatin remodelling and gene transcription. In addition to intranuclear functions, histones can be harmful when they enter the extracellular space.^27^ The administration of exogenous histones to animals leads to systemic inflammatory and toxic reactions. Animals treated with various anti‐histone preparations are protected from lethal endotoxaemia, sepsis, ischaemia, reperfusion injury, injury, pancreatitis, peritonitis, stroke, coagulation and thrombosis. In addition, elevated concentrations of histones and nucleosomes in the blood affect several pathophysiological processes and the progression of diseases such as AIPs, inflammation and cancer.^27^ We have recently shown that anti‐histone IgG in EAE mice effectively hydrolyses all five histones (H4, H3, H2a, H2b and H1).^22^ Thus, extracellular histones can, in principle, together with MOG or MBP, induce the development of AIPs, including EAE in mice and MS in humans.

Past studies have demonstrated that abzymes against a certain protein, as a rule, only hydrolyse this specific protein and no other globular control protein.^1^, ^2^, ^3^, ^4^, ^5^, ^6^ Hydrolysis of MBP is an intrinsic property of Abs in the blood of MS, SLE and HIV‐infected patients.^4^, ^28^, ^29^, ^30^, ^31^ At the same time, auto‐Abs of HIV‐infected patients^28^ and EAE mice^22^ can effectively hydrolyse all five histones. Until now, there are no data on abzymes cross‐hydrolysing any proteins against other proteins.^1^, ^2^, ^3^, ^4^, ^5^, ^6^, ^32^ Recently, it has been shown that in the blood of HIV‐infected patients, Abs against MBP effectively hydrolyse not only MBP but also histones and vice versa.^32^ It is believed that virus infection in humans leads to an accumulation of Abs against components of certain viruses that may have structural similarity to components of human blood and cells.^30^, ^31^, ^32^ Then, because of the mimicry of certain viral and human proteins, the immune system may fail, and Abs against components of the human body may be accumulated, resulting in the development of AIPs. According to our data, the beginning of AIP development may be associated not only with the mimicry of antigens but also with the homology between certain human proteins. For example, during cell apoptosis, nuclear histones with sequences homologous to MBP and other human proteins enter the blood. It is possible that the existence of cross‐catalytic activity of abzymes against histones and MBP is one of the internal causes of MS induction and other AIPs.^32^

Many SLE anti‐DNA Abs are directed against nucleosomal DNA‐histone complexes entering circulation after internucleosomal cleavage during apoptosis.^33^ It has been shown that polyclonal abzymes hydrolysing DNA of SLE and MS patients are cytotoxic, enter the nucleus and cause DNA fragmentation, inducing cell death by apoptosis.^34^, ^35^ In MS and SLE, anti‐MBP abzymes with protease activity can attack MBP of the myelin‐proteolipid sheath of axons.^28^, ^29^ Thus, these abzymes can play an important negative role in the pathogenesis of SLE and MS pathologies. However, auto‐Abs and abzymes hydrolysing other histones could also play a negative role in the pathogenesis of MS.^5^

Taking current knowledge into account, we wanted to understand which auto‐Abs without catalytic activity and which of the various abzymes demonstrating different levels of catalytic activity are produced at different EAE stages before and after immunization of mice. In this study, we, therefore, compared the levels of anti‐MOG, anti‐MBP, anti‐DNA and anti‐histone antibodies in different phases of EAE development (onset, acute and remission phases) before and after immunization of mice with MOG, DNA‐histone complex or DNA‐met‐BSA complex. In parallel, we determined the catalytic activity of abzymes hydrolysing DNA, MOG, MBP and histones and estimated correlations between levels of Abs and their ability to hydrolyse these substrates.

## 2 MATERIALS AND METHODS

### 2.1 Reagents and animals

If not stated otherwise, all chemicals, proteins and five histones (equimolar mixture of H4, H3, H2a, H2b and H1), bovine polymeric DNA, Protein G‐Sepharose and Superdex 200 HR 10/30 column were obtained from Sigma‐Aldrich (Munich, Germany). MOG_35‐55_ was obtained from EZBiolab (Germany), and human MBP (18.5 kDa) was purchased from the Research Center of Molecular Diagnostics and Therapy **(**RCMDT, Moscow, Russia). These preparations were free from nucleic acids, lipids, oligosaccharides and other possible contaminants.

C57BL/6 inbred mice (3 months of age) were kept in conditions free of viral and bacterial pathogens at the Institute of Cytology and Genetics (ICIG SB RAS) vivarium. All procedures with mice were done in accordance with the protocols of the Ethical Committee of the ICIG SB RAS, implementing the recommendations of the European Committee on Humane Principles of Working with Experimental Animals (Council Directive 86/609/CEE). The Ethics Committee of the ICIG SB RAS approved this study in accordance with Council Directive 86/609.

### 2.2 Immunization of mice and analysis of different parameters of EAE development

Immunization of C57BL/6 mice with MOG,^19^, ^20^ DNA‐met‐BSA complex^21^ and DNA‐histone complex^22^ was described previously (for details, see Supplementary methods). Analysis of various parameters during different phases of EAE development, including differentiation profiles of bone marrow HSCs and lymphocyte proliferation in different organs, was described previously^19^, ^20^, ^21^, ^22^ (for details, see Supplementary methods). In previous work^19^, ^20^, ^21^, ^22^ and in this work, parameter changes before and after immunization were analysed in groups of seven mice each.

### 2.3 ELISA of anti‐DNA, anti‐MOG and anti‐protein antibodies

In this study, we analysed previously collected samples concerning levels of Abs to different antigens of IgG preparations^19^, ^20^, ^21^, ^22^ at different time‐points of spontaneous and antigen‐induced development of EAE, which were not used before, and obtained additional data from frozen (−70^o^C) blood plasma preparations for a more detailed and advanced analysis.^19^, ^20^, ^21^, ^22^ The levels of anti‐DNA Abs (plasmas were diluted 100‐fold) were determined using standard ELISA: plates with immobilized double‐stranded DNA, and horseradish peroxidase–conjugated mouse Abs against human IgG of the test system ORGENTEC Diagnostika (Germany) were used as in Ref. 22 according to the manufacturer's instructions .

The relative levels of anti‐MBP and anti‐MOG IgGs were estimated according to Ref. 19, 20, 21, 22 For analysis of anti‐MOG, anti‐MBP Abs, plasmas were diluted 50‐fold using sodium carbonate buffer, pH 9.6. Diluted plasmas (50 μl) were added to ELISA strips, which were incubated overnight at 23°C. The strips were washed with TBS buffer (20 mmol/L Tris‐HCl containing 0.15 mol/L NaCl) supplemented with 0.01% NaN_3_ and 0.05% Triton X‐100 and three times with the same buffer containing no Triton X‐100. To block the strip surfaces, they were treated for 2.5 hours at 30°C using TBS containing 0.2% egg albumin and 0.01% NaN_3_. The strips were washed 8 times with water and then with TBS containing 0.01% NaN_3_. The strips were incubated with 100 μL of TBS containing 1 μg/mL conjugate of monoclonal anti‐human IgGs with horseradish peroxidase for 40 minutes at 30°C rewashed 10 times with water. After adding 60 μL citric‐phosphate buffer containing 3,3',5,5'‐tetramethylbenzidine, and H_2_O_2_, the strips were incubated for 14 minutes at 23°C, and the reaction was stopped by the addition of 6 μL of 50% H_2_SO_4_.

A_450_ values were determined using a UNISKAN II Plate Reader (MTX Lab Systems). The relative concentrations of Abs to MOG, MBP, DNA and histones were expressed as a difference in relative optical density (units A_450_; an average of 3 measurements) between experimental and control samples. Controls with MOG, MBP, DNA and histones, but without serum samples and without Abs interacting with the antigens, gave the same results.^22^

### 2.4 IgG purification

Electrophoretically homogeneous mouse IgG was purified by two consecutive chromatographies of plasma proteins on Protein G‐Sepharose, followed by FPLC gel filtration on Superdex 200 HR 10/30 column in dissociating conditions (pH 2.6), as described previously.^19^, ^20^, ^21^, ^22^ Abs were filtered through filter units (Millex; 0.1 μm) and stored in sterilized tubes to protect them from bacterial and viral contamination.^19^, ^20^, ^21^, ^22^ Electrophoretic homogeneity of IgG was analysed by SDS‐PAGE 4%‐15% gradient gels using non‐reducing conditions in the absence of DTT (0.1% SDS). IgG was revealed with silver staining gels, as described previously.^19^, ^20^, ^21^, ^22^ To exclude possible artefacts because of DNase and protease contaminations, IgG was separated by SDS‐PAGE, and its activities were revealed using a gel assay.^19^, ^20^, ^21^, ^22^ After electrophoresis, peaks for all activities were revealed only in those bands corresponding to intact IgG, and no other peaks were found for any canonical enzyme activities. More detailed data are given in previous work^19^, ^20^, ^21^, ^22^ and Supplementary methods.

### 2.5 DNA‐hydrolysing activity assay

DNase activity of IgG was analysed as described in previous work.^19^, ^20^, ^21^, ^22^ The reaction mixture (20 μL) contained 20 mmol/L Tris‐HCl (pH 7.5), 20 mmol/L NaCl, 5 mmol/L MgCl_2_, 1 mmol/L ethylenediaminetetraacetic (EDTA), supercoiled (sc) pBluescript DNA (20 μg/mL) and 0.03‐0.2 mg/mL of IgG. The mixture was incubated for 1‐12 hours at 37°C, and then, DNA hydrolysis was analysed using electrophoresis in 0.8% agarose gel. Ethidium bromide–stained gel was analysed with Gel‐Pro Analyzer v9.11.^19^, ^20^, ^21^, ^22^ The relative DNase activity was appraised from the percentage of the initial band of intact scDNA and its relaxed form, considering the relative content of DNA between these two bands for scDNA incubation in the absence of Abs. The products were analysed by looking at linear parts of rate dependencies of substrate hydrolysis on IgG concentrations and the time course (15%‐40% of DNA hydrolysis). The complete transformation of scDNA to its nicked form was taken as 100% of the activity. The RA (% of the hydrolysis) was finally recalculated to the same standard conditions.

### 2.6 Protease activity assay

The reaction mixtures (10‐30 μL) for analysing protease activities of IgG contained 20 mmol/L Tris‐HCl, pH 7.5; 0.7‐1.0 mg/mL of either MOG, MBP or equimolar mixture of five histones, and 0.01‐0.2 mg/mL of IgG, as described in detail in previous work.^19^, ^20^, ^21^, ^22^ They were incubated for 1‐21 hours at 37°C. The cleavage products of the proteins were analysed by SDS‐PAGE using 4%‐15% gradient or 12% gels. Gels were stained with Coomassie R250, scanned and then quantified using Gel‐Pro v3.1 software. The RAs of different IgGs were calculated from a decrease in the percentage of original proteins turned to their various hydrolysed forms. The hydrolysis of all proteins incubated without Abs was taken into account. Other measurements similar to the analysis of DNA hydrolysis were taken using pseudo‐first‐order reaction conditions.

### 2.7 Statistical analysis

The analysed parameters are given as the mean ± SD over three to four independent experiments, and data for each group are averaged over seven different mice. Correlation coefficients (CCs) between different parameters were estimated using the Pearson parametric method of Microsoft Excel 2010.

## 3 RESULTS

In several earlier studies, we analysed changes in the differentiation profile of bone marrow HSCs and the level of lymphocyte proliferation after immunization of C57BL/6 mice with MOG, DNA‐histone complex or DNA‐met‐BSA complex.^19^, ^20^, ^21^, ^22^ For illustration, previously obtained data are given in the Supplementary results. We evaluated changes in weight and proteinuria levels during spontaneous or antigen‐stimulated EAE development (Figure S1).^19^, ^20^, ^21^, ^22^ One of the important indicators for AIP development is an increase in mouse urine protein concentration.^36^ Until day 63, after immunization, the proteinuria of mice treated with MOG increased 3.8‐fold, whereas for mice treated with a complex of DNA‐histone or DNA‐met‐BSA, it decreased ~1.3‐fold (Figure S1). A decrease in proteinuria may slow down EAE development in mice after treatment with antigens containing DNA.^19^, ^20^, ^21^, ^22^

Figure S2 demonstrates that during spontaneous EAE development, the relative number of colonies changing overtime was very different for erythroid burst‐forming units, early erythroid colonies (BFU‐E); erythroid burst‐forming units, late erythroid colonies (CFU‐E); granulocyte‐macrophage colony‐forming units (CFU‐GM); and granulocyte, erythroid, myeloid colony‐forming units (CFU‐GEMM). Immunization of mice with MOG, DNA‐histone complex or DNA‐met‐BSA complex had completely different effects on the differentiation of HSCs (the relative content of BFU‐E, CFU‐E, CFU‐GM and CFU‐GEMM colonies) (Figure S2).^19^, ^20^, ^21^, ^22^

It was previously shown that the production of auto‐Abs and abzymes is associated not only with a change in differentiation profiles of HSCs but also with an increase in the level of lymphocyte proliferation (sum of T and B cells).^19^, ^20^, ^21^, ^22^ During spontaneous EAE development, we observed a constant increase in the relative content of lymphocytes in mouse bone marrow, spleen and thymus over time; a maximal increase was observed in bone marrow. Only for lymph nodes, we observed a remarkable decrease in the level of lymphocyte proliferation (Figure S3). Now, we have, for the first time, shown that EAE development can be accelerated not only by immunization of mice with MOG but also by treatment of them with DNA‐histone complex or DNA‐met‐BSA complex. In addition, we found that after treating mice with antigens containing DNA, the acute phase of EAE occurs much later than after MOG treatment. However, all three antigens change the differentiation profile of bone marrow stem cells and the proliferation of lymphocytes in various organs, accelerating the development of EAE associated with the production of auto‐Abs and abzymes hydrolysing MOG, MBP, DNA and histones. Abzymes with various enzymatic activities are the earliest and statistically most significant markers of AIP onset and development in humans and mammals,^2^, ^3^, ^4^, ^5^, ^6^ including human SLE,^29^, ^37^, ^38^ MS^15^, ^24^, ^25^, ^26^, ^28^, ^38^, ^39^ and experimental EAE in mice.^16^, ^17^, ^18^, ^19^, ^20^, ^21^, ^22^ Thus, during the development of various AIPs, both auto‐Abs without and abzymes with catalytic activities can be produced. Therefore, our main interest was to analyse the relative content of Abs against MOG, MBP, DNA and histones, and those abzymes harmful for mammals hydrolysing these substrates during spontaneous and antigen‐stimulated EAE development.

In this study, we used the following four groups of C57BL/6 mice, which differed in some of the parameters characterizing their EAE development, as described previously^19^, ^20^, ^21^, ^22^ and in the Supplementary results:

1. untreated control mice^19^, ^20^, ^21^, ^22^;
2. MOG immunized mice^19^, ^20^;
3. DNA‐met‐BSA complex immunized mice^21^;
4. DNA‐histone complex immunized mice.^22^

Some data on levels of Abs and their relative catalytic activity were taken from previous work.^19^, ^20^, ^21^, ^22^ On top, we obtained some additional data by analysing levels and relative activities of IgG using supplemental blood plasma preparations and IgG isolated from these preparations, according to procedures described previously.^19^, ^20^, ^21^, ^22^

New additional IgG preparations were purified from sera of individual mice by chromatography of unfrozen plasma proteins on Protein G‐Sepharose, followed by FPLC gel filtration.^19^, ^20^, ^21^, ^22^ All IgG preparations were electrophoretically homogeneous and, according to the analysis of the catalytic activities of gel fragment extracts after IgG SDS‐PAGE,^19^, ^20^, ^21^, ^22^ did not contain canonical DNase and protease enzymes.

### 3.1 Analysis of different parameters characterizing spontaneous EAE

As shown in previous work, autoimmune C57BL/6 mice reveal slow and spontaneous changes in different parameters, which are also relevant during EAE pathology, with onset as early as three months after birth.^19^, ^20^, ^21^, ^22^ Therefore, it is possible to reliably test hydrolysing properties of IgG derived from the blood of three‐month‐old mice for MOG, MBP, DNA and histones.^19^, ^20^, ^21^, ^22^

**Figure 1:**
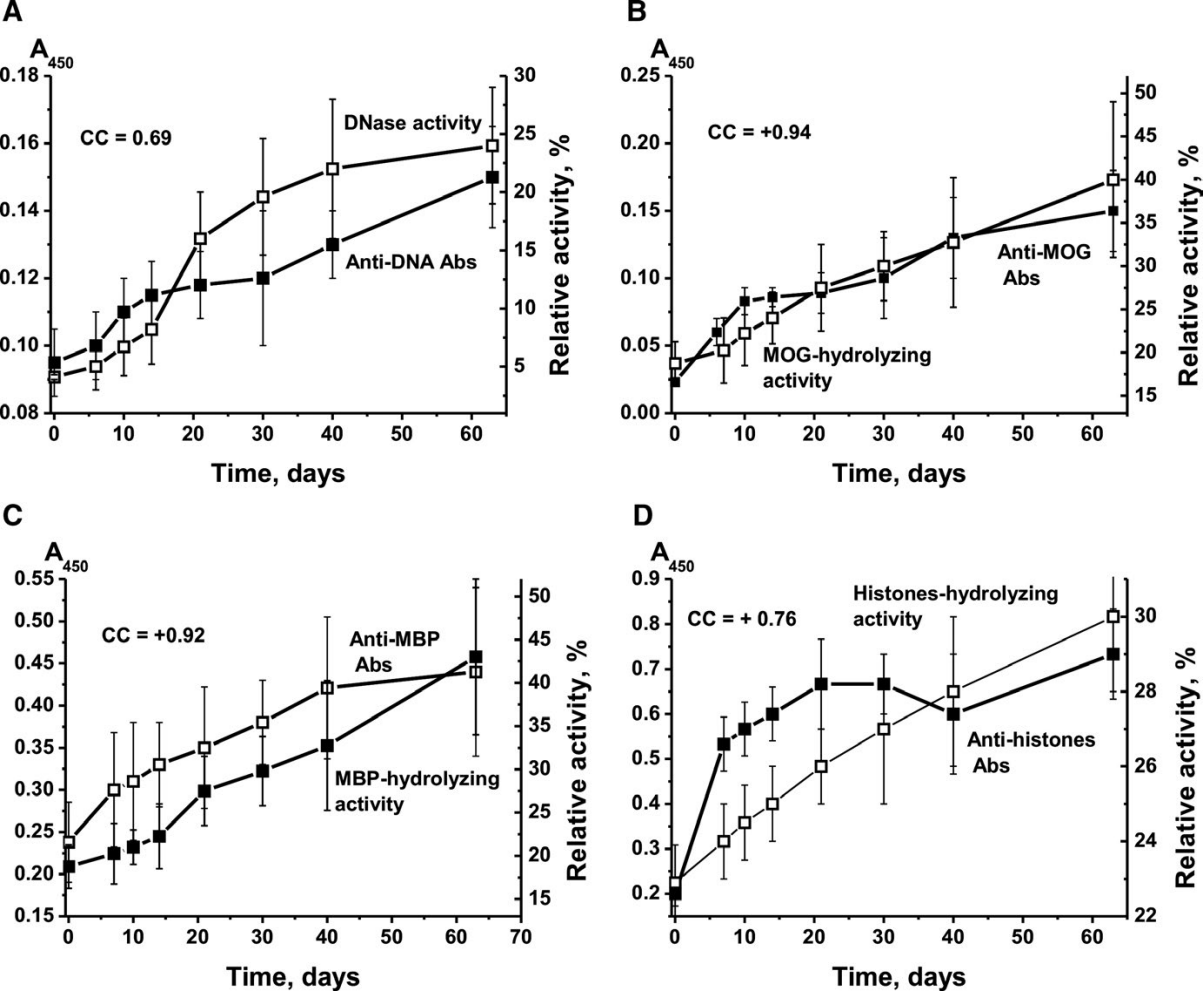
Time‐dependent changes, prior to immunization, in relative concentration of anti‐DNA Abs and relative activity (RA) of IgG hydrolysing DNA (A); of anti‐MOG antibodies and RA of IgG hydrolysing MOG (B); of anti‐MBP antibodies and RA of IgG hydrolysing MBP (C); and of anti‐histone antibodies and RA of IgG hydrolysing histones (D). The relative levels of Abs against DNA, MOG and MBP were evaluated using blood sera, whereas against five histones—electrophoretically homogeneous IgG. RA of histone hydrolysis corresponds to the average RA that characterizes the hydrolysis of the five histones H1, H2a, H2b, H3 and H4

During the 63‐day course of spontaneous EAE development, we observed a constant increase in the relative concentration of lymphocytes in the bone marrow and other organs of mice (Figure S3). However, over the same period, the blood concentration of Abs against DNA (increase 1.6‐fold), MBP (1.8‐fold), histones (3.7‐fold) and MOG (8.2‐fold) increased relatively slowly (Figure 1). The RA of IgG‐dependent hydrolysis, for preparations isolated from serum and levels of Abs to different antigens, DNA (CC = +0.69), MOG (+0.94), MBP (0.92) and histones (0.76), increased in parallel, with high CCs between relative concentrations of Abs and enzymatic activities of IgG (Figure 1). The presence of Abs against MOG and of abzymes hydrolysing this antigen prior to immunization indicates that the blood of mice contains myelin oligodendrocyte glycoprotein or its fragments, including MOG_35‐55_. In addition, already at three months of age, the blood of non‐immunized mice contains MBP or its fragments corresponding to the antigenic determinants of this protein. It is on these proteins or their fragments that the production of auto‐Abs with and without catalytic activity can occur.

### 3.2 Parameter analysis after immunization of mice with different antigens

Immunization of mice with MOG, DNA‐histone complex or DNA‐met‐BSA complex, and the parallel change in differentiation profile of bone marrow HSCs and increase in lymphocyte proliferation led to various changes in catalytic activities of Abs.

**Figure 2:**
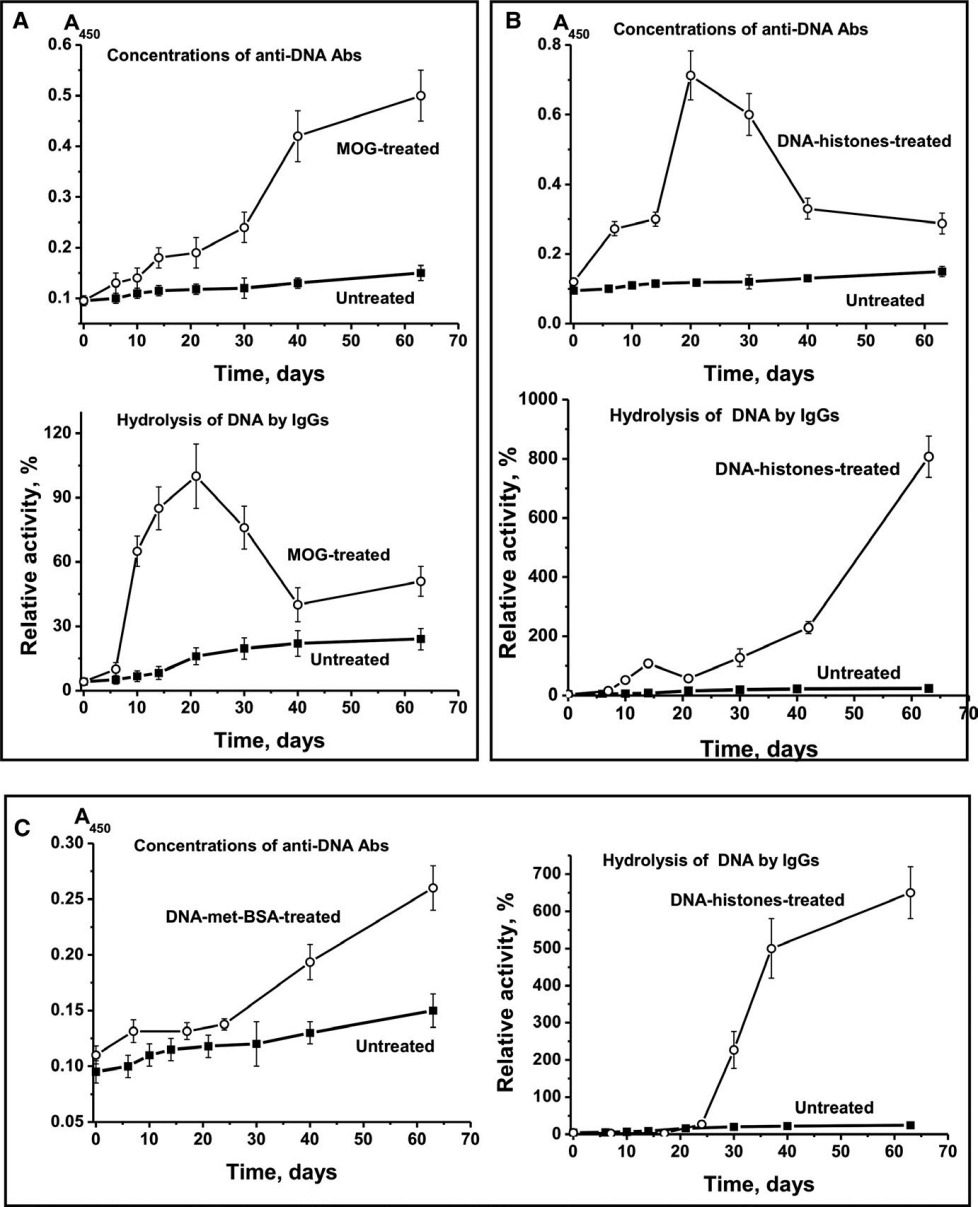
Time‐dependent changes in average values of anti‐DNA Ab concentration and relative activity of IgG hydrolysing DNA after immunization of mice with MOG (A), DNA‐histone complex (B) and DNA‐met‐BSA complex (C). For comparison, data on parameter changes during spontaneous development of EAE are given

Immunization of mice with MOG led to the hydrolysis of DNA by abzymes mainly in the period between onset of EAE development and exacerbation of the pathology (days 7‐20; Figure 2A). A significant increase in anti‐DNA antibody levels was observed after 30 days only and was associated with a sharp decrease in the DNase activity of abzymes (Figure 2A). CC between the relative concentration of anti‐DNA Abs and DNase activity was + 0.1.

**Figure 3:**
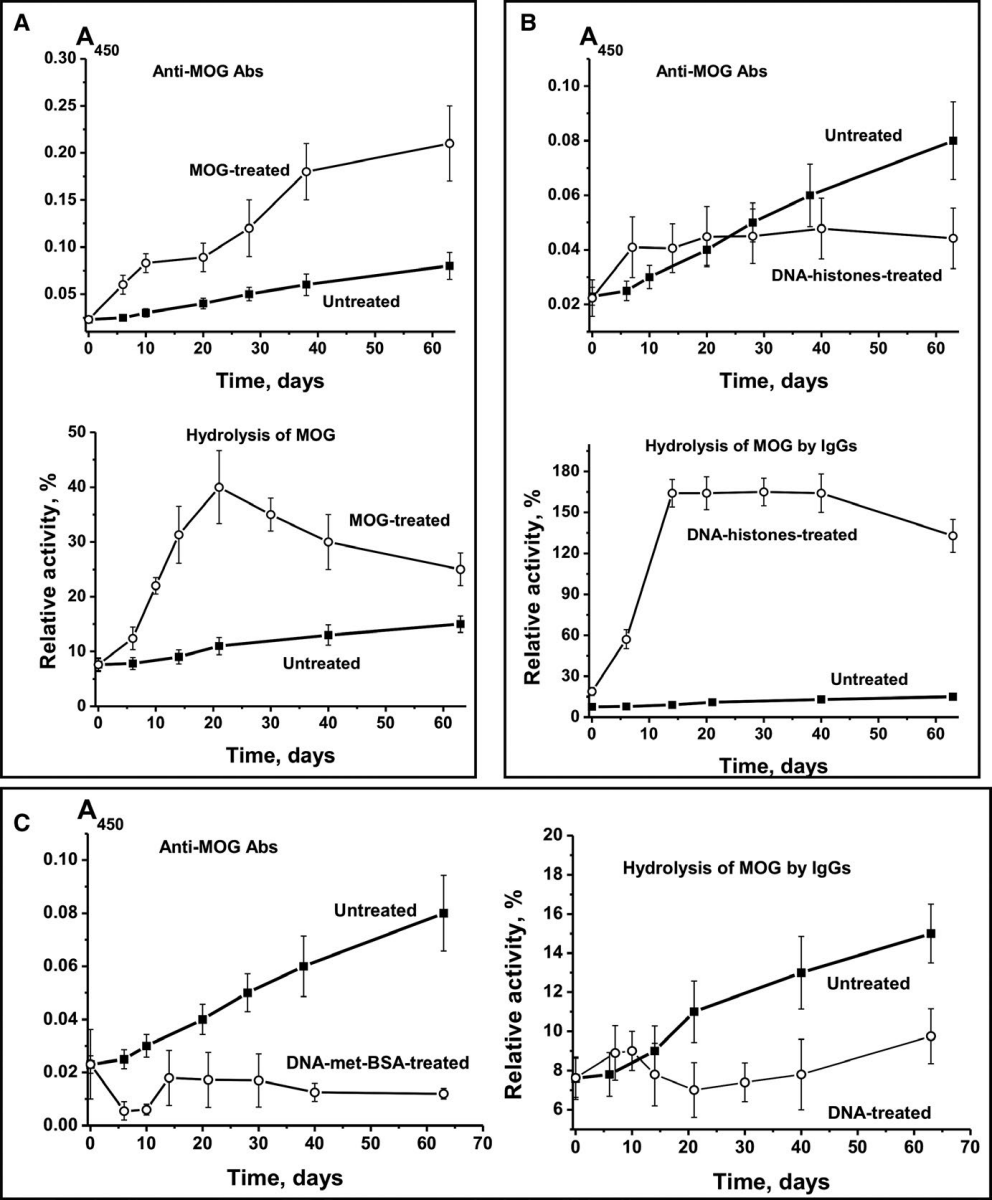
Time‐dependent changes in average values of anti‐MOG Ab concentration and relative activity of IgG hydrolysing MOG after immunization of mice with MOG (A), DNA‐histone complex (B) and DNA‐met‐BSA complex (C). For comparison, data on parameter changes during spontaneous development of EAE are given

**Figure 4:**
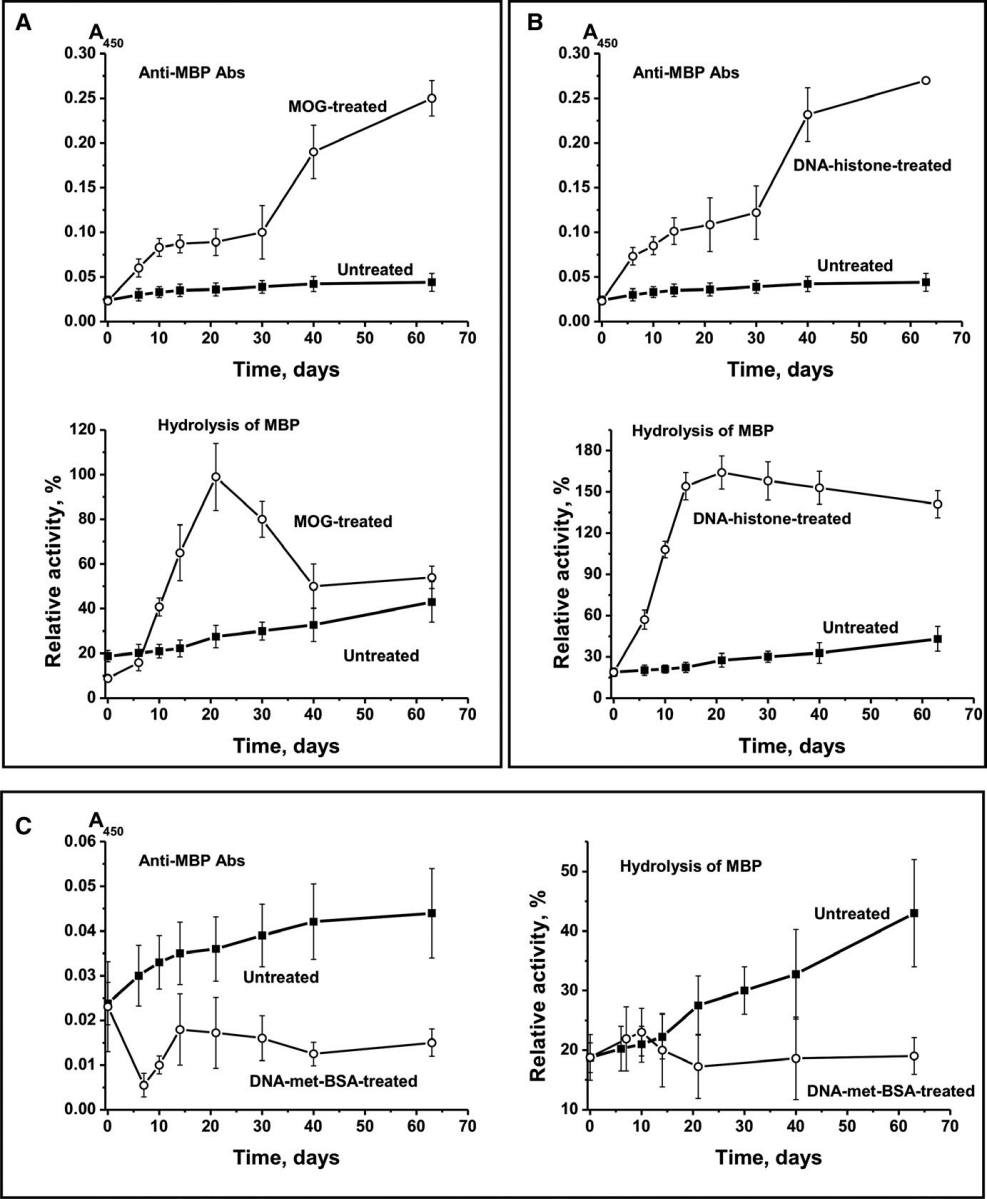
Time‐dependent changes in average values of anti‐MBP Ab concentration and relative activity of IgG hydrolysing MBP after immunization of mice with MOG (A), DNA‐histone complex (B) and DNA‐met‐BSA complex (C). For comparison, data on parameter changes during spontaneous development of EAE are given

After immunization of mice with MOG, CCs between anti‐MOG Abs and RA of IgG hydrolysing MOG (Figure 3A; CC = +0.47) and between anti‐MBP Abs and efficiency of MBP cleavage by IgG (Figure 4A; CC = +0.28) were moderate. Treating mice with MOG resulted in a similar increase in levels of antibodies to DNA (Figure 2A), MOG (Figure 3A) and MBP (Figure 4A). High positive CCs between Abs against different Abs were observed: anti‐DNA‐anti‐MOG (+0.98), anti‐DNA‐anti‐MBP (+0.97) and anti‐MOG‐anti‐MBP (+0.98). The RAs of IgG hydrolysing DNA, MBP and MOG also increased in parallel, with high CCs: DNA‐MBP (+0.97), DNA‐MOG (+0.98) and MBP‐MOG (+0.98). Thus, after treating mice with MOG, we observed a parallel synchronized activation of the production of Abs against DNA, MOG and MBP and abzymes hydrolysing these substrates. However, the relative level of Abs against DNA, MOG and MBP and the activity of IgG hydrolysing these substrates correlated only low or moderately (CCs = +0.28 to +0.46). This is because of abzymes being produced mainly during the onset and the acute phase of EAE (days 7‐20), whereas a significant increase in Ab levels against antigens occurred mostly during the remission period (days 20‐63) (Figure 3A,B). Hence, during remission, mostly anti‐DNA Abs without catalytic activity were produced.

It is obvious that complexes of DNA with histones and with met‐BSA should compete with intrinsic mouse myelin oligodendrocyte glycoprotein and its MOG oligopeptide for interaction with lymphocytes. Immunization of mice with DNA‐histone complex stimulated a significant increase (6‐fold) in the concentration of Abs against DNA after 21 days (Figure 2B). In contrast to immunization with MOG, the DNA‐histone complex stimulated only a small enhancement of DNase activity within the same period (Figure 2B). Subsequently, the concentration of anti‐DNA Abs sharply decreased (days 30‐63), whereas the activity of IgG‐dependent hydrolysis of DNA increased by 202‐fold compared with activity on day 0. The negative CC between the relative level of anti‐DNA Abs and DNase activity was very low, −0.09.

After immunization of mice with DNA‐histone complex, the concentration of Abs against MOG increased up to day 7, while during days 7‐63, it remained almost unchanged (Figure 2B). Hence, the level of Abs against MOG on day 63 was 1.8‐fold lower than at the onset of spontaneous EAE development (Figure 2B). At the same time, a small increase in the relative concentration of anti‐MOG Abs (2.2‐fold) led to an increase in IgG activity by a factor of 8.5 during days 7‐40. CC between the relative concentration of anti‐MOG Abs and MOG‐hydrolysing activity was + 0.82. This means that with a relatively small increase in anti‐MOG Abs (2.2‐fold), the DNA‐histone complex mainly stimulated the production of MOG‐hydrolysing abzymes (8.5‐fold).

After treating mice with DNA‐histone complex, the production of Abs against MBP followed a curve similar to the one corresponding to the production of Abs against MBP after treating mice with MOG (CC = +0.99) (Figure 4A,B). But the production of MBP Abs (Figure 4B) differed from the production of MOG Abs after treating mice with DNA‐histone complex (Figure 3B). After incubation with DNA‐histone complex, the level of antibodies against MOG increased up to day 7 and did not change thereafter (Figure 3B), whereas the concentration of Abs against MBP increased until day 63 (Figure 4A). However, the patterns of growth in the relative activity of IgG‐dependent hydrolysis of MOG and MBP after treating mice with DNA‐histone complex were very similar (Figure 3B and Figure 4B). After immunization with DNA‐histone complex, CC characterizing anti‐MBP Abs and IgG‐dependent hydrolysis of MBP was + 0.61. CC corresponding to growth of Abs against MOG and MBP was only + 0.62, whereas for the increase in the catalytic activity of IgG hydrolysing MOG and MBP it was + 0.99. Thus, after immunization of mice with MOG and DNA‐histone complex, there was some co‐ordination in the production of Abs against both MOG and MBP and the abzymes that hydrolyse these substrates.

We used the positively charged methylated BSA, forming strong complexes with DNA, as a protein that has DNA complex properties similar to the DNA complex with histones. However, the blood of C57BL/6 mice predisposed to spontaneous EAE development contains its own DNA complexes with histones, which can stimulate the production of auto‐Abs against DNA and histones. Therefore, it is obvious that intrinsic mouse DNA complexes with histones and DNA‐met‐BSA complex should compete for interaction with lymphocytes. DNA‐met‐BSA complex nearly completely suppressed the accumulation of Abs against MOG and the production of MOG‐hydrolysing abzymes observed in spontaneous EAE development (Figure 3C). However, a slight decrease in levels of anti‐MOG antibodies at 7‐10 days led to an increase in the activity of IgG hydrolysing MOG (Figure 3C): CC between these values was − 0.69. DNA‐met‐BSA complex also suppressed the development of Abs against MBP and the hydrolysis of this substrate by IgGs (Figure 4C); the CC between these values was also negative, −0.66. At the same time, the CC between levels of Abs against MOG and MBP (+0.96) and between IgG‐dependent hydrolysis of MOG and MBP (+0.63) was positive and high. Interestingly, after immunization of mice with DNA‐met‐BSA complex, similar to treatment with MOG and DNA‐histone complex, there was a distinct interconnection between production of Abs against MOG and MBP and abzymes hydrolysing these substrates.

### 3.3 Analysis of anti‐histone Abs after immunization of mice with different antigens

**Figure 5:**
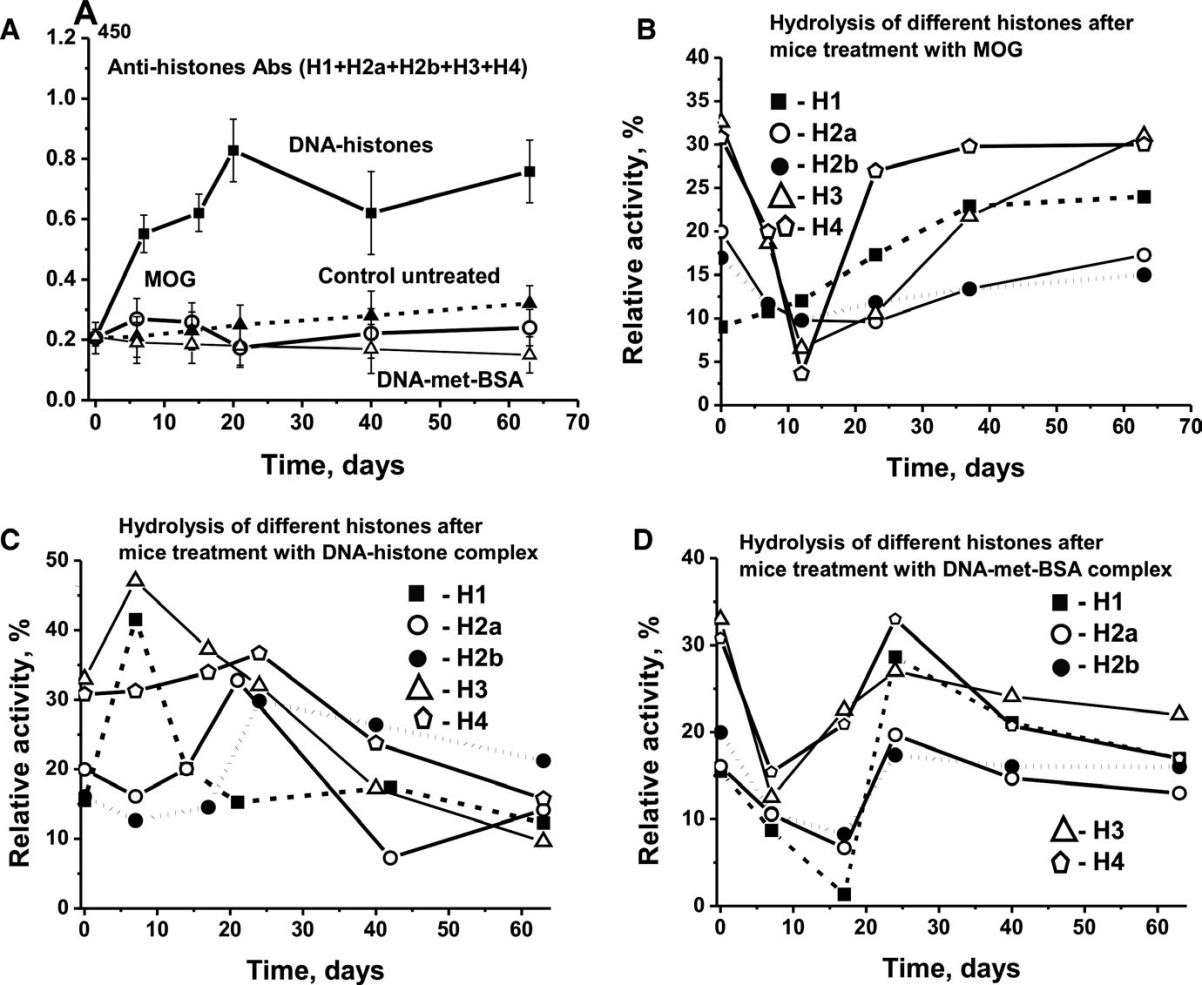
Time‐dependent changes in average values of anti‐histone Ab concentration (A) and relative activity of IgG hydrolysing five different histones (H1, H2a, H2b, H3 and H4) after immunization of mice with MOG (B), DNA‐histone complex (C) and DNA‐met‐BSA complex (D). The error in determining the value corresponding to each time‐point does not exceed 10%‐12%

The main antigen for which Abs against DNA is accumulated is a complex of DNA with histones.^33^ Histones can also play an important role in the development of AIPs.^27^ ELISA analysis of the relative concentration of Abs against the five histones (H1, H2a, H2b, H3 and H4) was carried out using their equimolar mixture (Figure 5A). During spontaneous EAE development, the relative concentration of Abs against five histones increased only 1.5‐fold by day 63. After immunization of mice with MOG, the concentration of Abs first slightly increased and then decreased. The treatment of mice with DNA‐met‐BSA complex stimulated a constant decrease in the concentration. The level of Abs only rose strongly (3.7‐fold) when mice were immunized with DNA‐histone complex (Figure 5A).

At all stages of EAE development, the efficiency of hydrolysis of each of the five histones was evaluated separately.^22^, ^31^, ^32^ After treating mice with MOG, we observed constant growth of protease activity only for hydrolysis of H1 histones, which increased 2.5‐fold by day 63 compared with time‐point zero (Figure 5B). MOG stimulated decrease in efficiency of IgG‐dependent hydrolysis of H2a, H2b, H3 and H4 in the period between 7 and 20 days, after which the activity of abzymes hydrolysing these four histones increased (Figure 5B).

The DNA‐histone complex induced the production of abzymes hydrolysing H1 and H3 histones on day 7, and for H2a, H2b and H4 at about 20 days after immunization. Thereafter, the activity of abzymes against all histones decreased significantly.

After immunization of mice with DNA‐met‐BSA complex, we observed very similar patterns (Figure 5D). The level of IgG‐dependent hydrolysis of all five histones was greatly reduced at 7‐14 days, and then sharply increased at about 20 days, with a strong decrease in the activities between 30 and 63 days. Overall, the synthesis of abzymes hydrolysing histones showed the tendency to decrease in the period following the initial 20 days after immunization, corresponding to the period of EAE remission.

It should be noted that the raise in Ab levels against the immunogens used and the hydrolysis activity of the abzymes differ strongly; there are both positive (from + 0.13 to + 0.91) and negative correlations (from − 0.09 to − 0.98).

## 4 DISCUSSION

We have previously demonstrated that changes in the differentiation profile of bone marrow HSCs and in lymphocyte proliferation in different organs lead to EAE development in C57BL/6 mice, associated with the production of auto‐Abs to MOG, MBP, DNA and histones and abzymes—harmful for mice—hydrolysing these substrates.^19^, ^20^, ^21^, ^22^ Immunization of mice with MOG, DNA‐histone complex or DNA‐met‐BSA complex accelerates EAE development compared with spontaneous disease progression. In parallel, we observe various changes in the differentiation profiles of HSCs (Supplementary results) and in the synthesis of different auto‐Abs and abzymes.^19^, ^20^, ^21^, ^22^

It is believed that auto‐Abs against body's own antigens may be harmful for individuals, and their levels increase greatly with the development of various AIPs.^1^, ^2^, ^3^, ^4^, ^5^, ^6^ However, Abs‐abzymes hydrolysing human antigens can be more harmful and are important in AIP pathogenesis. DNase abzymes are cytotoxic; they penetrate the nucleus of cells and stimulate cell death because of apoptosis.^34^, ^35^ Anti‐MBP and anti‐histone abzymes can hydrolyse MBP of the myelin‐proteolipid sheath of axons^28^, ^29^, ^32^ and therefore may also be important for the development of human MS and EAE in animals.

In this study, we are the first to analyse in detail the possible ratio of auto‐Abs against MOG, MBP, histones and DNA without activity and abzymes hydrolysing these antigens during the different stages of spontaneous and antigen‐induced EAE. Particularly notable about our data is the powerful increase in catalytic abzyme activity accompanied by either a strong decrease or an increase in auto‐Ab levels. To analyse our results, it is necessary to consider some data from the literature.

One of the features of abzymes involved in catalytic activities in different AIPs is their extreme diversity and heterogeneity (for review, see^38^). The exceptional diversity of all polyclonal abzymes with nuclease activities, derived from the blood of patients with AIPs,^37^, ^38^, ^39^, ^40^ autoimmune mice^41^ and animals immunized with DNA, RNA, RNase A, DNase I and DNase II,^4^, ^5^, ^6^ was demonstrated by data of total IgG chromatography on DNA cellulose. When using a concentration gradient of NaCl (0.05‐3 mol/L), IgG and its catalytic activity were distributed across its chromatography profile. Some of the DNase abzymes were eluted from the column only by applying 2‐3 mol/L MgCl_2_ or an acidic buffer (pH 2.3).

A further study showed the distribution of abzymes derived from the blood of MRL‐lpr/lpr mice for fractions with different affinities to DNA and different relative activities in the presence of Mg^2+^, Mn^2+^ and Ca^2+^.^41^ A similar significant distribution of Abs and their activity across the entire profile occurs during chromatography of total IgG derived from the blood of patients with MS or SLE on Sepharose with immobilized MBP or other proteins.^29^ All Ab fractions eluted from the sorbent differed in their relative catalytic activities and other properties. Interestingly, depending on the patient, as well as the specific disease and its duration, the repertoire of Abs hydrolysing DNA, RNA, proteins, oligosaccharides and other antigens may be relatively small or very broad.^1^, ^2^, ^3^, ^4^, ^5^, ^6^, ^38^ In the later stages of AIPs, the total pool of immunoglobulins usually contains Abs with kappa‐ and lambda‐type light chains. Abzymes can catalyse various reactions at different pH values, dependent or not on different metal ions. IgG of four subclasses (IgG1‐IgG4) with protease activity can be serine, thiol, acidic or metalloproteases. Their contribution to total abzyme activities depends on the patient, individual AIP, specific substrate and other factors.^1^, ^2^, ^3^, ^4^, ^5^, ^6^, ^38^

One study looked at the cDNA library of only kappa light chains of Abs from patients with SLE (Ref. 38 and references therein). Only 45 of 451 individual colonies, corresponding to a single peak eluted from DNA cellulose with 0.5 mol/L NaCl, and 33 of 687 colonies of the peak, eluted with an acidic buffer, were analysed. In the first case, 15 out of 45 homogeneous preparations of monoclonal light chains (MLChs; ~ 33%) and, in the second case, 19 out of 33 MLChs (58%) demonstrated DNase activity.^38^

To analyse MBP‐hydrolysing activity, 72 of 440 individual colonies, corresponding to the peak eluted from the MBP‐Sepharose with 0.5 mol/L NaCl, were used; 25 of 72 MLChs (~ 35%) effectively hydrolysed MBP. Kappa MLChs of all 10 peaks eluted from DNA cellulose and MBP‐Sepharose actively hydrolyse DNA and MBP, respectively.^38^ If we consider the average percentage of active monoclonal abzymes (~42%) in any of 10 eluted peaks, a possible number of kappa‐abzymes with DNase and MBP‐hydrolysing activity in 10 peaks can be ≥1000. At the same time, polyclonal Abs from the blood of patients with SLE or MS, with DNA‐ and MBP‐hydrolysing activities, contain kappa and lambda light chain.^2^, ^3^, ^4^, ^5^, ^6^ All polyclonal and monoclonal abzymes have very different RAs (from very low to very high) for hydrolysing various substrates. Taking into account the exceptional heterogeneity and extreme diversity of abzymes, each AIP stage may be accompanied by the synthesis of many different Abs without catalytic activity and abzymes with very different relative catalytic activities hydrolysing various antigens. These data from the literature are useful for understanding our results, which reveal the production of very different auto‐Abs with and without catalytic activity at different stages of EAE development in C57BL/6 mice.

We here demonstrate that the concentration of auto‐Abs against DNA, MOG, MBP and five histones during spontaneous EAE development gradually increased in parallel with the increase in activity of abzymes hydrolysing these substrates; CCs between concentrations of auto‐Abs and their catalytic activities varied from + 0.69 to + 0.94 (Figure 1). Immunization of mice with MOG, DNA‐histone complex or DNA‐met‐BSA complex, and thereby accelerating EAE development, dramatically changes the situation. For example, the synthesis of abzymes with high MOG‐ and MBP‐hydrolysing activities occurred during the onset and acute phases of EAE after immunization of mice with MOG (days 6‐20), when the Ab levels against MOG and MBP were relatively low; they increased between 20 and 63 days, which was associated with the production of IgG with low or without activity (Figure 3A and Figure 4A). At the same time, mice immunized with the DNA‐histone complex in the initial and acute phases (days 6‐20) demonstrated a high level of anti‐DNA Abs, whereas the activity of the abzymes was relatively low (Figure 2B). The subsequent ~2.5‐fold decrease in the anti‐DNA concentration between 20 and 63 days after immunization with the DNA‐histone complex is accompanied by a 200‐fold increase in abzyme activity (Figure 2B). More than 20 days after treating mice with the DNA‐met‐BSA complex, an increase in both levels of anti‐DNA Abs and DNase activity of abzymes occurred (Figure 2C). However, abzyme RA increase (290‐fold from day 1 to 63) is 52 times higher than the increase in anti‐DNA Ab levels (5.6‐fold). Thus, in both cases, immunization of mice with antigens containing DNA led to a strong delay in the production of specific anti‐DNA abzymes with high catalytic activity (maximum activity at 63 days) compared with the treatment of mice with MOG (maximum activity at 20 days); there is a 6.5‐fold difference in DNase activity (Figure 2). Any of the antigens used accelerated EAE development. However, EAE development after immunization of mice with MOG or with antigens containing DNA proceeded in different ways. These antigens (especially MOG and the DNA‐met‐BSA complex) are, to some extent, antagonists, stimulating different changes in the differentiation profiles (Supplementary results) and production of auto‐Abs without and with catalytic activities during EAE development.

Overall, our study is the first showing that immunization of mice with different antigens (MOG, DNA‐histone complex or DNA‐met‐BSA complex) causes various changes in the differentiation profiles of HSCs, associated with significant differences in the synthesis of auto‐Abs against MOG, MBP, histones and DNA. At the same time, each stage of EAE development after immunization of mice with MOG and DNA complexes is characterized by a specific ratio of auto‐Abs without activities and abzymes hydrolysing MOG, MBP, DNA and histones. It is entirely unexpected that immunization with each antigen (MOG, DNA‐histone complex or DNA‐met‐BSA complex) has a completely different effect on the time course of the production of IgG hydrolysing the five histones: H1, H2a, H2b, H3 and H4 (Figure 5).

All three immunogens accelerate EAE development in mice. Immunization of mice with MOG leads to a powerful parallel production of Abs against MOG, MBP and DNA and abzymes hydrolysing these substrates during initial and acute EAE phases (days 6‐20). In contrast, after treating mice with antigens containing DNA, there is a big delay in the synthesis of DNA‐hydrolysing Abs (days 30‐63). However, at 63 days, IgG‐dependent efficiency of DNA hydrolysis is about 6.5‐8.0 times higher after treatment with antigens containing DNA than the maximal DNase activity of IgG after immunization of mice with MOG.

## CONFLICT OF INTEREST

The authors declare no conflicts of interest.

## AUTHOR CONTRIBUTIONS

IAO, NAP, VNB, GAN, conception and design of the experiments. KSA, AAU, SES, LBT, JAL, conduction of experiment. GAN, TB, SGM, IAO, NAP, data analysis. IAO, NAP, SVS, TB, SGM, contribution of reagents/materials/analysis tools. GAN, TB, SGM, IAO, writing of the manuscript. GAN, TB, SGM, development of theoretical description.

## Supporting information

Fig S1‐S3

File S1
