# Supplementary material for: The association between EAE development in mice and the production of autoantibodies and abzymes after immunization of mice with different antigens
Source: J Cell Mol Med. 2021 Feb 9;25(5):2493–504. doi: 10.1111/jcmm.16183 (PMC7933958; doi:10.1111/jcmm.16183)
Supplement: Supplementary file 1 — Fig S1‐S3 [file JCMM-25-2493-s001.docx]

**Fig. S1.** Relative changes over time in parameters characterizing C57BL/6 EAE mice: body weight (**A**); proteinuria (**B**). Data were taken from previous work [19-22].

**Comments to Figure S1**

Weight changes of C57BL/6 mice treated with DNA-histones were analyzed for 63 consecutive days starting from day of immunization (at three months of age, time point zero). Control experiments with untreated mice and animals treated with MOG and with DNA-met-BSA were performed in parallel. Data of these experiments are similar to previously published results [19-20], indicating that the conditions were well reproducible. In contrast to untreated and MOG-treated mice, but similar to mice treated with DNA-met-BSA complex (decrease of weight compared to control 1.2-fold; *p* < 0.05), immunization with DNA-histones led to an essential weight decrease (1.1-fold; *R* < 0.05) during the 63-day observation period.

Prior to immunization with antigens, C57BL/6 mice demonstrated high proteinuria (up to 8–12 mg/ml) even at three months of age. While proteinuria increased nearly gradually from day 0 to 63 from 7.2 to 27.8 mg/ml in MOG-treated mice, it almost did not change in untreated control mice until day 20, and then gradually increased to 17.0 mg/ml by day 63. After treating mice with DNA-met-BSA, proteinuria did not markedly change during the first 30 days, and then slightly increased to 9.4 mg/ml by day 63. Urine protein levels of mice immunized with DNA-histones first decreased until day ~25, but then increased and became comparable to levels seen for the DNA-met-BSA complex 30–63 days after immunization [19-22].

**Fig. S2.** Changes over time in mouse brain BFU-E (**A**), CFU-E (**B**), CFU-GM (**C**), and CFU-GEMM (**D**) colony-forming units are shown for untreated mice, and after treatment with MOG, DNA-histones complex, or DNA-met-BSA complex. Immunogens used are shown on Panels **A-D**.

**Comments to Figure S2**

We analyzed changes over time in four types of colonies: erythroid burst-forming unit, early erythroid colonies (BFU-E); erythroid burst-forming unit, late erythroid colonies (CFU-E); granulocytic-macrophagic colony-forming unit (CFU-GM); and granulocytic, erythroid, myeloid colony-forming unit (CFU-GEMM) [19-22]. Data on changes over time in mouse brain colonies were well reproducible in four independent studies [19-22]. Inducing spontaneous EAE development with various antigens resulted in completely different differentiation profile characteristics for mouse bone marrow stem cells. More detailed information can be found in previous work [22].

**Fig. S3.** The average changes over time in optical density, reflected by relative amount of lymphocytes in bone marrow (**A**), spleen (**B**), thymus (**C**), and lymph nodes (**D**), are shown for untreated mice, and after treatment with MOG, DNA-histones complex, or DNA-met-BSA complex. Immunogens used are shown on Panels **A-D**. The error in optical density estimation for each mouse for all groups (with seven mice per group) from three independent experiments did not exceed 7–10%.

**Comments to Figure S3**

For C57BL/6 mice, production of abzymes is associated not only with a change in HSC differentiation profiles but also with an increase in lymphocyte proliferation levels (sum of T and B cells) [19-22]. During spontaneous EAE development, relative levels of lymphocyte proliferation in bone marrow, spleen, and thymus constantly increased over time, with a maximal increase observed in bone marrow. Only for lymph nodes the level of lymphocyte proliferation decreased. Treating mice with either of the three antigens resulted in dramatic changes in lymphocyte proliferation, with very distinct patterns of change for bone marrow, spleen, and thymus. More detailed information can be found in previous work [22].
