# Supplementary material for: The association between EAE development in mice and the production of autoantibodies and abzymes after immunization of mice with different antigens
Source: J Cell Mol Med. 2021 Feb 9;25(5):2493–504. doi: 10.1111/jcmm.16183 (PMC7933958; doi:10.1111/jcmm.16183)
Supplement: Supplementary file 2 — File S1 [file JCMM-25-2493-s002.docx]

**Supplementary methods**

**Immunization of mice**

C57BL/6 mice were immunized with MOG and DNA-met-BSA as described in previously published protocols [19-21]. On day 1 (time point zero), mice were immunized by injecting 10 μg of MOG into the back of each mouse via two injections in the left and right side using 20 μl of complete Freund’s adjuvant containing Pertussis toxin (400 ng/mouse; *Mycobacterium tuberculosis*)*.* On the following day, an additional 20 μl of Pertussis toxin (400 ng/mouse) was injected in a similar way [19].

EAE mice were immunized twice with 40 µg of polymeric thymus DNA that was conjugated with methylated BSA and dissolved in physiological solution, as described previously [21]. A mixture of 0.5 volume of complete Freund’s adjuvant and 0.5 volume of antigen solution was used. The mixture was stirred to achieve a homogeneous gel and injected subcutaneously or into the paw pads. The second immunization, with a mixture containing incomplete Freund’s adjuvant, was performed after 2 days.

C57BL/6 mice were immunized with a gel (150 µl) combining polymeric DNA complex (94 µg), five histones (92 µg) and Pertussis toxin (0.4 µg) [22]. To prepare a conjugate, a solution of 23.6 mg five histones (in 11.8 ml of water) was mixed with 23 mg calf thymus DNA (in 3 ml of water), adding 80 μl of 3 M NaOH (pH 10). After complete dissolution the mixture was titrated with 1 M hydrochloric acid to pH 7.0 and diluted to 18.8 ml with a physiological solution containing 0.235 M NaCl. As final step, 18.8 ml of antigen solution, 18.8 ml complete Freund’s adjuvant solution and 101.5 µg Pertussis toxin (*Mycobacterium tuberculosis)* in 20 µl of water were mixed. This mixture was repeatedly passed through a syringe needle to form a homogeneous gel. Per mouse, 100 µl of the gel were injected subcutaneously; 50 µl into each paw pad. The second immunization of each mouse (after 2 days) was performed in the same manner using a 150 µl mixture of incomplete Freund’s adjuvant containing 0.4 µg Pertussis toxin.

The relative weight of mice and levels of proteinuria (relative concentration of total protein in the urine, mg/ml) were analyzed as described previously [19-22]. Protein concentration in urine was measured using the Bradford assay with a bovine serum albumin standard. For further experiments, including the purification of Abs and analysis of their enzymatic activity, 0.5-0.8 ml of blood were collected after decapitation using standard approaches [19-22].

**Analysis of bone marrow progenitor cells in culture**

Bone marrow samples were flushed out from mouse femurs, and the colony-forming ability of the bone marrow cells was estimated, as described in previous work [19-22]. Four dishes per mouse (each containing 2×10^4^ cells) were cultured in a standard methylcellulose-based M3434 medium specific for mouse cells (StemCell Technologies, Canada). The medium contained stem cell factor, interleukin (IL)-3, IL-6, and erythropoietin (EPO). Relative number of CFU-GM, CFU-E, BFU-E, and CFU-GEMM colonies were calculated after 14 days of sample incubation at 37^o^C and 5% CO_2_ in a humidified incubator, as described previously [17-21].

**Analysis of lymphocyte proliferation**

Analysis of lymphocyte proliferation *in vitro* (sum of B and T cells) was carried out, as described previously [19-22]. Cells (10^6^/ml) isolated from spleen, bone marrow, lymph nodes, and thymus were cultivated in 96-well flat-bottom plates (Trasadingen, Switzerland) containing RPMI-1640 medium supplemented with 10 mM HEPES buffer, 10% fetal calf serum, 2 mM L-glutamine, 0.5 mM 2-mercaptoethanol, 100 μg/ml benzylpenicillin, and 80 μg/ml gentamicin. After a 64-hour incubation period, a solution (15 μl) containing 5 mg/ml MTT (tetrazolium dye MTT is 3-(4,5-[di](http://en.wikipedia.org/wiki/Di-)[methyl](http://en.wikipedia.org/wiki/Methyl)[thiazol](http://en.wikipedia.org/wiki/Thiazole)-2-yl)-2,5-di[phenyl](http://en.wikipedia.org/wiki/Phenyl)tetrazolium bromide) was added to each well and plates were incubated at 37^o^C for an additional 4 h. Then, plates were centrifuged for 10 min at 1200×g and solutions were removed. Cells were precipitated by adding DMSO (200 μl); the mixtures were resuspended and incubated at 23^o^C for 15 min in darkness. The analysis of the relative cell amount was performed spectrophotometrically at 492 nm.
